# Altered levels of sphingolipid metabolites in serum of locally advanced rectal cancer patients: A pilot study

**DOI:** 10.5937/jomb0-55113

**Published:** 2025-06-13

**Authors:** Jasna Bjelanović, Aleksandra Nikolić, Mutay Aslan, Marko Miladinov, Nikola Kotur, Goran Barišić, Sandra Dragicević

**Affiliations:** 1 University Clinical Center of Serbia, Center for Medical Biochemistry, Belgrade; 2 University of Belgrade, Institute of Molecular Genetics and Genetic Engineering, Belgrade; 3 Akdeniz University Medical Faculty, Department of Biochemistry, Antalya, Turkey; 4 University Clinical Center of Serbia, Clinic for Digestive Surgery-First Surgical Clinic, Belgrade; 5 University of Belgrade, Faculty of Medicine, Belgrade

**Keywords:** apoptosis, neoadjuvant chemoradiotherapy, rectal cancer, sphingolipids, apoptoza, neoadjuvantna hemioradioterapija, karcinom rektuma, sfingolipidi

## Abstract

**Background:**

Altered sphingolipid levels might contribute to rectal cancer development, progression and therapy response by regulating various biological processes, including apoptosis. This study aimed to analyse the serum sphingolipid profile in rectal cancer patients and investigate its association with the apoptotic status of tumour tissue and therapy response.

**Methods:**

Ceramide (CER) and sphingomyelin (SM) serum levels were analysed in 22 patients with locally advanced rectal cancer and 24 healthy individuals by ultrafast liquid chromatography coupled with tandem mass spectrometry. The expression of pro-apoptotic BAX (BCL2 associated X, apoptosis regulator) and anti-apoptotic BCL2 (BCL2 apoptosis regulator) was analysed in tumour and corresponding healthy tissue samples of patients by quantitative real-time PCR.

**Results:**

Significantly lower serum levels of C18 CER, C22 CER, C24 CER, C18 SM and C24 SM were observed in patients than in controls (P<0.05). For C20 CER, C22 CER and C24 CER, a positive correlation with the pro-apoptotic status of tumour tissue was found (r=0.619, P=0.018; r=0.694, P=0.006 and r=0.601, P=0.023, respectively). No difference in serum sphingolipid levels was found between patients with good, moderate, and poor responses to therapy.

**Conclusions:**

These results support the involvement of sphingolipids in rectal cancer. However, further studies, including a larger cohort of subjects, are needed to clarify the association of sphingolipid metabolites with therapy response.

## Abstract (sr)

**Uvod:**

Promene u nivou sfingolipida mogu da doprinesu nastanku karcinoma rektuma, progresiji bolesti i odgovoru na terapiju usled njihove regulacije različitih bioloških procesa uključujući apoptozu. Cilj ove studije je bio da se analizira profil sfingolipida u serumu kod pacijenata sa karcinomom rektuma i da se ispita njegova povezanost sa apoptotskim statusom tkiva tumora i odgovorom na terapiju.

**Metode:**

Nivo ceramida (CER) i sfingomijelina (SM) je analiziran u serumu kod 22 pacijenta sa lokalno uznapredovalim karcinomom rektuma i kod 24 zdrave osobe pomoću ultrabrze tečne hromatografije kombinovane sa tandem masenom spektrometrijom. Ekspresija pro-apoptotskog gena BAX (BCL2 asocirajući X, regulator apoptoze) i anti-apoptotskog gena BCL2 (BCL2 regulator apoptoze) je analizirana pomoću metode kvantitativni PCR u realnom vremenu u uzorcima tumorskog tkiva i zdrave crevne sluznice kod pacijenata.

## Introduction

According to GLOBOCAN 2022, colorectal cancer (CRC) is the third most common malignant disease and the second leading cause of cancer death in the world [1]. Approximately one-third of all colorectal carcinomas belong to rectal cancer, and the American Cancer Society reported 46.220 new cases of rectal cancer in 2024 [2]. Although malignant transformation occurs as a consequence of activation of oncogenes and/or inactivation of tumour suppressor genes, the onset and progression of CRC might also be associated with changes in cellular lipidome, including changes in fatty acids, phospholipid and sphingolipid composition [3] [4]. Since the cancer stage at the moment of diagnosis influences the length of survival and treatment options, various studies were conducted to identify CRC biomarkers in biological fluids. Among potential metabolic biomarkers, special attention was paid to sphingolipids [5]. However, the role of these molecules in rectal cancer has been understudied.

Sphingolipids are bioactive molecules that control cellular events such as signal transduction, cell growth, differentiation, and apoptosis. The central molecules in sphingolipid biosynthesis and catabolism are ceramides (CER). They consist of sphingosine attached to the long-chain fatty acids. The members of ceramide families differ depending on the fatty acid chain length. The most abundant mammalian sphingolipid is sphingomyelin (SM), obtained by adding phosphocholine to ceramide. Other complex sphingolipids are glycosphingolipids that contain one or more sugar residues attached to ceramide [6].

Ceramides could be synthesised *de novo* from serine and palmitoyl CoA or produced by hydrolysis of complex sphignolipids. They are further metabolised into sphingosine, and the degradation of ceramides represents the primary source of sphingosine [6] [7]. Ceramides and sphingosine are tumour-suppressor lipids that mediate growth inhibition, differentiation, and apoptosis [7]. On the other hand, their phosphorylated derivatives, ceramide-1-phosphate (C1P) and sphingosine-1-phosphate (S1P) exhibit tumour-promoting effects by promoting cell proliferation, migration, transformation, inflammation, and angiogenesis. Although sphingomyelins exhibit anti-tumour effects, it is still unclear whether they depend on increased hydrolysis and the formation of ceramides [3].

Previous studies investigating dysregulation of sphingolipid metabolism and cellular accumulation of sphingolipids in CRC mainly focused on colon cancer. The changes in ceramide content in colon cancer were suggested to be due to the altered level or activity of ceramide synthases [8]. Increased serum levels of specific ceramides and sphingosine were found in patients with CRC compared to controls [9]. It was also reported that higher tissue levels of ceramides positively correlate with advanced stages of CRC [8] [10]. Additionally, the association of long-chain ceramides with lymph node metastases was suggested. Ceramide synthases and sphingosine kinase are increased in colon cancer tissue compared to adjacent normal mucosa [8].

The implications of sphingolipid metabolites in response to CRC therapy have been investigated as well. It is supposed that de novo ceramide production is the mechanism involved in apoptosis induced by chemoradiotherapy (CRT) [7]. The ceramide generation and accumulation in response to therapy enhance apoptosis of tumour cells. The association of overexpressed C6 CER in colon cancer cells with increased susceptibility to 5-FU chemotherapy was found [11]. Additionally, ceramide plasma level in CRC metastatic patients was considered a predictor of response to radiotherapy in combination with irinotecan [8]. However, the accumulation of complex sphingolipids due to increased ceramide levels and their conversion into inert biomolecules is proposed to be associated with resistance to CRT [3].

Numerous studies analysed sphingolipid molecules and enzymes as biomarkers of progression and therapy response in colorectal cancer, where data from colon and rectal cancer patients were mixed. Our study aimed to analyse the serum sphingolipid profile in rectal cancer patients and investigate its association with the apoptotic status of tumour tissue and therapy response.

## Materials and methods

### Subjects

This study included 22 patients with locally advanced rectal cancer (LARC) who were diagnosed and treated at the Clinic for Digestive Surgery – First Surgical Clinic, Clinical Center of Serbia, between April 2019 and May 2020. The control group consisted of 24 healthy blood donors who entered the study between August 2019 and January 2020. The characteristics of the participants are given in Table 1.

**Table 1:** Characteristics of the study groups. LARC – locally advanced rectal cancer; SD – standard deviation; HDL – high–density lipoprotein; LDL– low–density lipoprotein; CRP – C-reactive protein; CEA – carcinoembryonic antigen; CA 19-9 – carbohydrate antigen 19-9; CA 72-4 – tumour-associated glycoprotein 72; CA 15-3 – cancer antigen 15-3; nCRT – neoadjuvant chemoradiotherapy; RCRG – Rectal Cancer Regression Grade

	LARC patients (n=22)	Controls (n=24)
Age (years), mean (SD)	65.2 (11.5)	46.0 (11.1)
Males, n (%)	15 (68.2)	11 (44.0)
Glucose (mmol/L), mean (SD)	6.3 (1.5)	4.9 (0.3)
Proteins (mmol/L), mean (SD)	72.8 (3.7)	73.1 (4.5)
Albumin (mmol/L), mean (SD)	43.9 (2.2)	44.5 (2.6)
Cholesterol (mmol/L), mean (SD)	5.6 (1.3)	5.0 (0.8)
HDL- Cholesterol (mmol/L), mean (SD)	1.3 (0.3)	1.3 (0.3)
LDL- Cholesterol (mmol/L), mean (SD)	3.5 (1.0)	3.0 (0.8)
Triglycerides (mmol/L), mean (SD)	1.5 (0.4)	1.4 (0.6)
CRP (IU/mL), mean (SD)	5.9 (6.4)	3.1 (3.9)
CEA (IU/mL), mean (SD)	15.9 (47.9)	2.6 (0.6)
CA 19-9 (IU/mL), mean (SD)	22.9 (56.8)	5.6 (5.2)
CA 15-3 (IU/mL), mean (SD)	13.9 (5.1)	15.5 (6.3)
CA 72-4 (IU/mL), mean (SD)	5.4 (6.1)	3.9 (6.4)
Stage at diagnosis		
T stadium, n (%)		
T3	14 (63.6)	-
T4	8 (36.4)	-
N stadium, n (%)		
N1	4 (18.2)	-
N2	18 (81.8)	-
Response to nCRT, n (%)		
RCRG1	3 (18.8)	-
RCRG2	7 (43.8)	-
RCRG3	6 (37.4)	-

Patients were subjected to tissue and serum sampling at two points, before and 8–12 weeks after treatment with neoadjuvant CRT (nCRT), a maximum of 48 hours preoperatively. At first sampling, primary tumour tissue and the adjacent healthy mucosa (pairs of tissue samples) were obtained by biopsy, while at second sampling, tumour and non-tumour tissue samples were obtained intraoperatively. Serum samples were collected from patients during routine testing before biopsy and surgery. All tissues and sera were immediately and adequately processed after sampling and stored at -80°C until further use.

In all patients, adenocarcinoma was histopathologically confirmed. According to the American Joint Committee on Cancer criteria, pretreatment staging was determined using the tumour-node-metastasis (TNM) system. The absence of metastases was evaluated using computed tomography (CT) and/or magnetic resonance imaging (MRI). The measurements of carcinoembryonic antigen (CEA), carbohydrate anti gen 19-9 (CA 19-9), cancer antigen 15-3 (CA 15-3) levels in serum samples were performed using Chemiluminescent Microparticle Immunoassay method, while tumour-associated glycoprotein 72 (CA 72-4) and C-reactive protein (CRP) were determined by Electrochemiluminiscence immunoassay and immunoturbidimetric assay, respectively. Routine biochemistry parameters: glucose, proteins, albumin and lipid profile (cholesterol, HDL-cholesterol, LDL-cholesterol and triglycerides) were measured by spectrophotometry. Control serum samples were subjected to the same analyses as patient samples.

Sixteen of 22 patients were subjected to nCRT followed by surgical resection. Preoperative treatment included a total dose of 50.4 Gy of irradiation in 28 fractions combined with two or three cycles of chemo - therapy (5-fluorouracil 425 mg/m2 and Leucovorin 20 mg/m^2^). All resected specimens were histopathologically examined, and tumour response to nCRT was assessed using Rectal Cancer Re gres sion Grade (RCRG). Based on the relative amount of tumour and fibrosis, tumour regression grade was classified as the following: RCRG1 – complete res ponse or only microscopic foci of carcinoma remaining, with marked fibrosis; RCRG2 - moderate res ponse with macroscopic disease present; RCRG3 – minimal response characterised by the predominance of tumour tissue or no response, without visible signs of tumour regression.

This study was conducted according to the guidelines of The Code of Ethics of the World Medical Association (Declaration of Helsinki) and approved by the Ethical Committee of the University Clinical Center of Serbia (447/6; October 19, 2021). Informed consent was obtained from all participants.

### Sphingolipid measurements by LC-MS/MS

Serum sphingolipids were measured by ultrafast liquid chromatography (UFLC; LC-20 AD UFLC XR, Shimadzu Corporation, Japan) coupled with tandem mass spectrometry (MS/MS; LCMS-8040, Shimadzu Corporation, Japan), as previously described [12]. Standards for N-palmitoyl-D-erythro-sphingosylphosphorylcholine (C16 SM), N-stearoyl-D-erythro sphingosylphosphorylcholine (C18 SM), N-lignoceroyl-D-ery thro sphingosylphosphorylcholine (C24 SM) N-palmitoyl-D-erythro-sphingosine (C16 CER), N-stearoyl-D-erythro-sphingosine (C18 CER), N-arachidoyl- D-erythro-sphingosine (C20 CER), N-behenoyl-D-erythro- sphingosine (C22 CER) and N-lignoceroyl- D-erythro-sphingosine (C24 CER) were purchased from Avanti Polar Lipids (Alabaster, USA). Labelled C16 CER d18:1/16:0 (Palmitoyl-U-13C16) internal standard was obtained from Cambridge Isotope Laboratories (Andover, USA). Responses to analysed sphingolipids were optimised to a linear calibration range with a sample analysis time of 35 min. Serum samples were prepared for LC-MS/MS analysis as previously described [13].

### Relative quantification of mRNA expression level

Total RNA was extracted from tissue samples using TRI Reagent Solution (Thermo Fisher Scientific, Lithuania) according to the manufacturer’s protocol. The RNA concentration and purity were determined by 260 nm and 280 nm absorption using a BioSpecnano spectrophotometer (Shimadzu Corporation, Japan). For mRNA expression analysis of target genes, two mg total RNA was reverse-transcribed using a High Capacity cDNA Reverse Transcription kit (Applied Biosystems, USA) according to the manufacturer’s protocol. The reaction conditions were 10 min at 25°C, 120 min at 37°C, and 5 min at 85°C.

The mRNA expression of target genes (*BCL2* - BCL2 apoptosis regulator and *BAX *- BCL2 associated X, apoptosis regulator) was measured in triplicate by quantitative Real-Time PCR (qRTPCR) using the Power SYBR™ Green PCR Master Mix (Applied Biosystems, USA). Melting curve analysis was performed for all reactions to validate the specificity of the products. Actin beta (*ACTB*) was used as an internal housekeeping gene control for all experiments. The primer sequences and length of the products were the following: *BCL2 *forward 5’-TCGCCCTGTGGATGACTGA- 3’ and *BCL2 *reverse 5’- CAGAGACAGCCAGGAGAAATC- 3’ (134bp); *BAX *forward 5’- TGGCAGCTGACATGTTTTCTGAC-3’ and *BAX *reverse 5’-TCACCCAACCACCCTGGTCTT-3’ (195bp); *ACTB *forward 5’-GGACTTCGAGCAAGAGATGG-3’ and *ACTB *reverse 5’-AGGAAGGAAGGCTGGAAGAG- 3’ (138bp).

The qRTPCR was performed on 7500 Real- Time PCR System (Applied Biosystems, USA). The reaction conditions were 2 min at 50°C, 10 min at 95°C followed by 40 cycles of 15 s at 95°C, and 1 min at 60°C. The relative expression of target genes was normalised to the expression of the housekeeping gene and calculated by applying the 2^-dCt^ method.

### Statistical analysis

Statistical analysis was performed by Statistical Package for Social Sciences 20.0 (SPSS Inc., Chicago, Illinois, USA). Categorical variables are presented as frequencies, while continuous variables are expressed as means with standard deviation (SD). The Shapiro-Wilk test was used to assess the normality of continuous data. Depending on the data distribution, differences between independent samples were analysed by the Independent-Sample t-test, Mann-Whitney U and Kruskal-Wallis tests, while differences between matched samples were analysed by the Paired Sample t-test and Related Samples Wilcoxon signed-rank test. The degree of association between variables was calculated using parametric Pearson’s correlation coefficient (r) and a non-parametric Spearman’s rank correlation coefficient (r_s_). A p-value of less than 0.05 was considered statistically significant.

## Results

### Analysis of sphingolipid content

This study included 22 LARC patients (mean age 65.2 (11.5), 68.2% were males) and 24 healthy individuals (mean age 46.9 (11.3), 44.0% were males). There was no significant difference in the values of basic biochemical parameters between groups.

The profiles of ceramides and sphingomyelins were analysed in serum samples obtained from all subjects by LC-MS/MS. Among ceramides, the highest levels were observed for C24 CER, followed by C22 CER and C16 CER in both groups. The level of C20 CER was higher than C18 CER in patients, while in the control group, it was the opposite. The most abundant sphingomyelin in the serum of patients and controls was C16 SM, followed by C18 SM and C24 SM.

**Figure 1:**
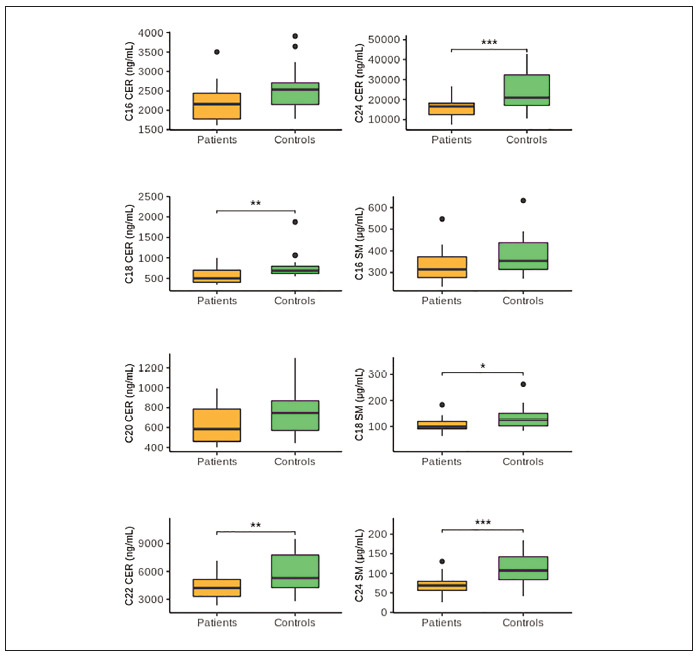
The serum levels of ceramides and sphingomyleins in LARC patients compared to controls. Sphingolipid concentrations are presented using boxes, with the horizontal line inside the box representing the median value and lower and upper whiskers representing the minimum and maximum value. Differences between groups were analysed by Independent-Sample t-test (normal distribution of data) except for C16 SM, C18 SM and C18 CER, where the Mann-Whitney U test (non-normal data distribution) was used. LARC – locally advanced rectal cancer; CER – ceramide; SM – sphingomyelin; **P<0.01; ***P<0.0005

To analyse the association of sphingolipids with rectal cancer, the observed serum levels of ceramides and sphingomyelins were compared between patients and controls (Figure 1). Of the ceramides analysed, a statistically significant difference was found for C18 CER (P=0.005), C22 CER (P=0.005), and C24 CER (P<0.0005). Lower serum levels of these sphingolipids were observed in patients than in controls. Although a similar trend was noticed for C16 CER and C20 CER, statistical significance was not achieved (P=0.056 and P=0.060, respectively). All analysed sphingomyelins were decreased in patients compared to controls, with the statistically significant difference found only for C18 SM and C24 SM (P=0.036 and P<0.0005, respectively).

The association of the ceramides and sphingomyelins with tumour markers and CRP was investigated (Table 2). The results showed a positive correlation between C18 SM and CRP (r=0.574, P=0.032). A similar trend was observed between C24 SM and CA19-9, with statistical significance near borderline (r=0.484, P=0.057).

**Table 2:** Correlation of serum sphingolipid profile with tumour and inflammatory markers, and with apoptotic score in LARC patients before treatment with nCRT. LARC – locally advanced rectal cancer; nCRT – neoadjuvant chemoradiotherapy; CEA – carcinoembryonic antigen; CA 19-9 – carbohydrate antigen 19-9; CA 72-4 – tumour-associated glycoprotein 72; CA 15-3 – cancer antigen 15-3; CRP – C-reactive protein; *BAX* – BCL2 associated X, apoptosis regulator; *BCL2 *– BCL2 apoptosis regulator; CER – ceramide; SM – sphingomyelin<br>r – Pearson’s correlation coefficient (normal distribution of data); rs – Spearman’s rank correlation coefficient (non-normal distribution of data); *P<0.05

	CEA (IU/mL)	CA19-9 (IU/mL)	CA15-3 (IU/mL)	CA72-4 (IU/mL)	CRP (IU/mL)	*BAX/BCL2* ratio
C16 CER <br>(ng/mL)	r_s_=0.059	r =-0.031	r=0.019	rs=0.399	rs=0.174	r =0.491
	P=0.834	P=0.910	P=0.956	P=0.199	P=0.553	P=0.075
C18 CER <br>(ng/mL)	r_s_=0.109	r_s_=0.209	r_s_=0.097	r_s_=0.336	r_s_=0.377	r_s_=0.293
	P=0.457	P=0.436	P=0.778	P=0.286	P=0.253	P=0.303
C20 CER <br>(ng/mL)	r_s_=0.116	r =0.332	r=-0.104	r_s_=0.266	r_s_=0.152	r=0.619
	P=0.680	P=0.209	P=0.761	P=0.404	P=0.605	P=0.018*
C22 CER <br>(ng/mL)	r_s_=-0.029	r =0.225	r=-0.056	r_s_=0.140	r_s_=0.029	r=0.694
	P=0.919	P=0.403	P=0.871	P=0.665	P=0.923	P=0.006**
C24 CER <br>(ng/mL)	r_s_=-0.045	r =0.180	r=-0.085	r_s_=-0.028	r_s_=-0.248	r=0.601
	P=0.874	P=0.505	P=0.804	P=0.931	P=0.392	P=0.023*
C16 SM <br>(μg/mL)	r_s_=0.333	r =0.079	r=-0.065	r_s_=0.399	r_s_=0.288	r=0.091
	P=0.225	P=0.770	P=0.850	P=0.199	P=0.318	P=0.757
C18 SM <br>(μg/mL)	r_s_=0.487	r =0.126	r=0.103	r_s_=0.483	r_s_=0.574	r=0.217
	P=0.066	P=0.642	P=0.764	P=0.112	P=0.032*	P=0.455
C24 SM <br>(μg/mL)	r_s_=0.365	r =0.484	r=0.410	r_s_=0.182	r_s_=-0.007	r=0.356
	P=0.181	P=0.057	P=0.210	P=0.572	P=0.982	P=0.212

**Figure 2:**
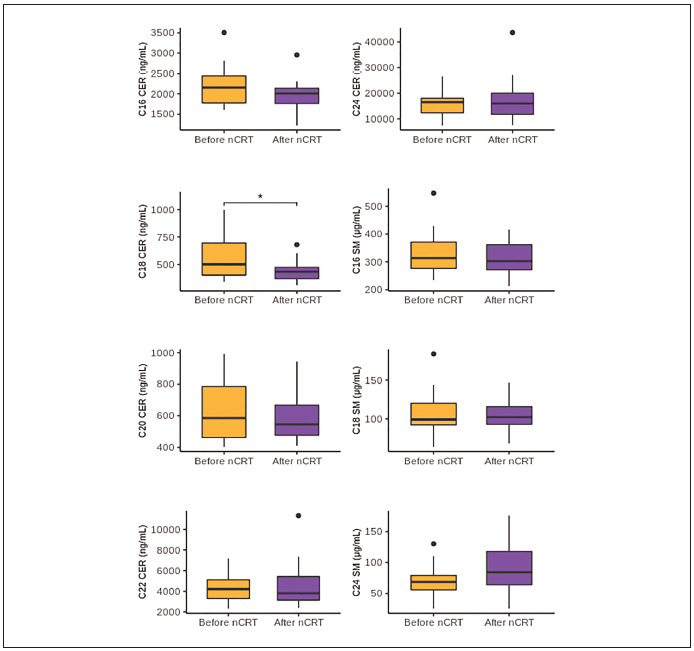
The serum levels of ceramides and sphingomyleins in LARC patients before and after treatment with nCRT. Sphingolipid concentrations are presented using boxes, with the horizontal line inside the box representing the median value and lower and upper whiskers representing the minimum and maximum value. Differences between samples were analysed by Paired Sample t-test and (normal distribution of data) except for C18 CER, C22CER, and C24 CER, where Related samples Wilcoxon signed-rank test (non-normal distribution of data). LARC – locally advanced rectal cancer; nCRT – neoadjuvant chemoradiotherapy; CER – ceramide; SM – sphingomyelin; *P<0.05

Sixteen patients were subjected to nCRT, and the sphingolipid content in serum samples collected from these patients was compared before and after treatment (Figure 2). Only for C18 CER was found to decrease after nCRT (P=0.031) significantly. To evaluate the association of sphingolipids with response to nCRT, patients were divided according to the pathological response into three groups: patients with good response - RCRG1 (n=3), moderate response - RCRG2 (n=7), and poor response - RCRG3 (n=6). The serum levels of ceramides and sphingomyelins were compared between groups. We found no association with the RCRG stage for any of the analysed sphingolipids (P>0.05). Also, we found no association of observed C18 CER decrease after nCRT with the pathological response (P=0.663).

### Analysis of apoptotic status of tissue samples

To determine the apoptotic status of tissue samples, the relative mRNA expressions of *BAX *as proapoptotic marker and *BCL2* as qRTPCR measured anti-apoptotic marker, and the *BAX/BCL2* ratio was calculated.

The BAX/BCL2 ratio association in tumour tissue samples with sphingolipid serum profile was investigated. Only serum levels of C20 CER, C22 CER and C24 CER were found to positively correlate with the *BAX/BCL2* ratio in tumour tissues in patients before undergoing nCRT (Table 2). With the increase of these ceramide species in serum, the *BAX/BCL2* ratio increases in tumour tissue. Additionally, we examined the association between the *BAX/BCL2* ratio and response to nCRT. However, no significant association was found (P>0.05).

## Discussion

Sphingolipids are considered essential for the structural integrity of the intestinal tract. Understanding sphingolipid metabolism in the colon and rectum is crucial for understanding its role in CRC development, progression and therapy response. Healthy individuals typically have a more balanced metabolic profile, whereas the altered metabolic state in CRC patients might reflect lipid metabolism changes distinct from controls. Alterations in the metabolism of sphingolipids may be caused by modification of endogenous enzymatic activity or the addition of dietary sphingolipids. The first step in dietary sphingomyelin metabolism is its hydrolysis into proapoptotic ceramide within the intestine lumen by alkaline sphingomyelinase. On the other hand, ceramides with distinct fatty acid chains are generated by six differentially expressed isoforms of ceramide synthase. Reduced generation of ceramides due to decreased alkaline sphingomyelinase or ceramide synthase might contribute to colon cancer development [14].

In our study, lower levels of all analysed ceramides and sphingomyelins were observed in the serum of LARC patients in comparison to healthy controls, with a statistically significant difference found for C18 CER, C22 CER, C24 CER, C18 SM and C24 SM. The patients included in the study were not subjected to any restrictive diet. Still, they consumed a diverse range of foods, providing a more comprehensive insight into the role of sphingolipids in rectal cancer, independent of dietary factors. Although dietary sphingolipid intake can influence circulating ceramide and sphingomyelin levels, the varied diet of the patients contributes to a more realistic depiction of their role in rectal cancer. Nevertheless, dietary habits remain an essential factor that should be further considered in future research.

Our finding that ceramide levels are decreased in the serum of LARC patients compared to healthy controls is opposite to the previously published research [9]. In normal tissue, the gradient in the expression and activity of alkaline sphingomyelinase from the colon to the rectum has been shown [14]. Our findings are also opposite to the study results that found upregulation of both ceramide and sphingomyelin (3% and 5%, respectively) in the serum of CRC patients compared to healthy controls [15]. This upregulation of both sphingolipid species was found in patients with metastatic CRC, while our study has included only LARC patients in whom metastases were excluded. A previous study found decreased concentrations of pro-apoptotic ceramide and increased concentrations of pro-proliferative S1P in polypoid lesions with high malignancy potential and the opposite in those with low malignancy potential [16]. Control of S1P levels in colon cancer cells depends on the up-regulation or down-regulation of the enzymes included in S1P synthesis or degradation, respectively. In the catabolic pathway, S1P might be irreversibly degraded or synthesised back to ceramide and other complex sphingolipids [14]. Other studies found ceramide levels to be increased in later stages of CRC [10].

The reduced ceramide concentrations observed in our study may be due to impaired levels or activity of acid ceramidase (ASAH1). This enzyme maintains the balance between pro-apoptotic and pro-proliferative sphingolipids [17]. ASAH1 hydrolyses ceramides into sphingosine, which can subsequently be phosphorylated into S1P, while ceramides can also undergo phosphorylation to generate C1P [3] [17]. Both S1P and C1P have been implicated in tumour progression, highlighting the importance of ASAH1 in regulating their availability. These facts make ASAH1 a key regulator of ceramide metabolism and an essential modulator of C1P and S1P levels. Given its central role in sphingolipid metabolism and potential impact on therapy response, further studies are needed to investigate ASAH1 expression and activity in rectal cancer patients to determine its association with tumour development and treatment outcomes.

*In vitro* findings contradict literature data on the association of ceramide and sphingomyelin levels with CRC. A decrease in sphingomyelin and ceramide lipid levels was observed in SW620 compared to the SW480 cell line [18]. Cell line SW480 is a primary source of SW620 isogenic lymph node metastasised derivative, and the migratory potential and invasiveness of SW480 cells are increased compared to SW620 cells [19] [20].

The decreased sphingolipid levels in LARC patients compared to healthy controls may be attributed to multiple factors. Sphingolipid content can be influenced by non-genetic factors, such as hypoxia [21]. It should also be noted that different studies used different sample types, and the plasma profile of sphingolipids is known to differ from the tissue [10].

Since alterations in sphingolipid levels and/or the activity of sphingolipid-metabolising enzymes could influence therapeutic response, our study explored ceramides and sphingomyelins in response to nCRT. Among the analysed sphingolipids, we observed a reduction in the expression of C18 CER. Considering that de novo ceramide synthesis has previously been suggested as a key mechanism involved in nCRT-induced apoptosis, our finding may result from impaired activity of ceramide synthase 1, which is responsible for C18 CER production [7] [22]. Further research should investigate the levels and activity of ceramide synthases and the activity of ASAH1, given that it has been shown to influence sensitivity to radiation therapy in CRC [23].

The role of sphingolipid metabolism in response to nCRT was evaluated only by a few studies. Combinations of C6 CER with adjuvant therapy elicited mitochondrial production of reactive oxygen species and cytochrome c release and induced apoptosis [24]. Resistance to 5-FU in DLD-1/5-FU colorectal cancer cells was mainly associated with increased sphingomyelin and decreased ceramide [25]. Additionally, colon cancer cells resistant to drug treatment exhibited high enzyme expression levels, including in converting ceramides into glycosphingolipids [14].

The study did not observe differences in sphingolipid levels between patients with different responses to nCRT. Considering our cohort’s limited number of responders, the association between sphingolipids and therapy response should be evaluated in a larger sample set.

We found that serum levels of C20 CER, C22 CER and C24 CER positively correlate with apoptotic status of tumour tissues. The ratio between pro-apoptotic BAX and anti-apoptotic BCL2 increases with the increase of these ceramides. A positive correlation of ceramides with the pro-apoptotic status of the tumour tissue might be expected, considering their tumour suppressive roles and involvement in apoptosis [25] [26] [27]. Previous *in vitro* studies showed that HCT116 colon cancer cells express proteins involved in cell death and growth arrest in response to ceramide treatment [14]. Our results also support previous findings that the influence of ceramides on the growth and survival of cancer cells depends on the length of the fatty acid chain [8]. Therefore, assessing the apoptotic status of tumour tissue based on the serum levels of specific ceramides might be proposed. We found no association between the *BAX/BCL2* ratio in tissue samples and response to nCRT. Considering that the majority of samples in this study had higher expression of BAX than BCL2 (*BAX/BCL2* ratio >1) and that there were only three responders to therapy (RCRG 1), this finding should be reevaluated in a larger cohort.

Unlike many similar studies that have investigated sphingolipid molecules in colon cancer, our study was focused exclusively on rectal cancer. Decreased serum levels of specific ceramide and sphingomyelin molecules in LARC patients were found, and a positive correlation of very long-chain ceramides (C20- C24) with tumour tissue pro-apoptotic status was found.

Considering these findings and the maintenance of low circulating levels of pro-apoptotic ceramides is associated with the survival of tumour cells, our preliminary results support the involvement of sphingolipid species in rectal cancer. However, further studies, including a larger cohort of subjects, are needed to clarify the association of sphingolipid metabolites with therapy response.

## Dodatak

### Acknowledgements

This work was supported by nthe Strategic Project of the Serbian Academy of Sciences and Arts, the Molecular Basis of Response to Chemoradiotherapy in Rectal Cancer – MOHERA - TEKA, F-69. The article is based upon work from COST action CA17118, supported by COST (European Cooperation in Science and Technology; www.cost.eu). The authors owe gratitude to Tuğçe Çeker and Çağatay Yılmaz for their technical assistance in the preparation of serum samples for LC-MS/ MS analysis.

### Conflict of interest statement

All the authors declare that they have no conflict of interest in this work.
